# A state-of-the-art review on coir fiber-reinforced biocomposites

**DOI:** 10.1039/d1ra00231g

**Published:** 2021-03-12

**Authors:** K. M. Faridul Hasan, Péter György Horváth, Miklós Bak, Tibor Alpár

**Affiliations:** Simonyi Károly Faculty of Engineering, University of Sopron Sopron Hungary k.m.faridul.hasan@phd.uni-sopron.hu alpar.tibor@uni-sopron.hu

## Abstract

The coconut (*Cocos nucifera*) fruits are extensively grown in tropical countries. The use of coconut husk-derived coir fiber-reinforced biocomposites is on the rise nowadays due to the constantly increasing demand for sustainable, renewable, biodegradable, and recyclable materials. Generally, the coconut husk and shells are disposed of as waste materials; however, they can be utilized as prominent raw materials for environment-friendly biocomposite production. Coir fibers are strong and stiff, which are prerequisites for coir fiber-reinforced biocomposite materials. However, as a bio-based material, the produced biocomposites have various performance characteristics because of the inhomogeneous coir material characteristics. Coir materials are reinforced with different thermoplastic, thermosetting, and cement-based materials to produce biocomposites. Coir fiber-reinforced composites provide superior mechanical, thermal, and physical properties, which make them outstanding materials as compared to synthetic fiber-reinforced composites. However, the mechanical performances of coconut fiber-reinforced composites could be enhanced by pretreating the surfaces of coir fiber. This review provides an overview of coir fiber and the associated composites along with their feasible fabrication methods and surface treatments in terms of their morphological, thermal, mechanical, and physical properties. Furthermore, this study facilitates the industrial production of coir fiber-reinforced biocomposites through the efficient utilization of coir husk-generated fibers.

## Abstract 2

The coir fibers could be used as prominent biocomposite materials.

## Graphical abstract

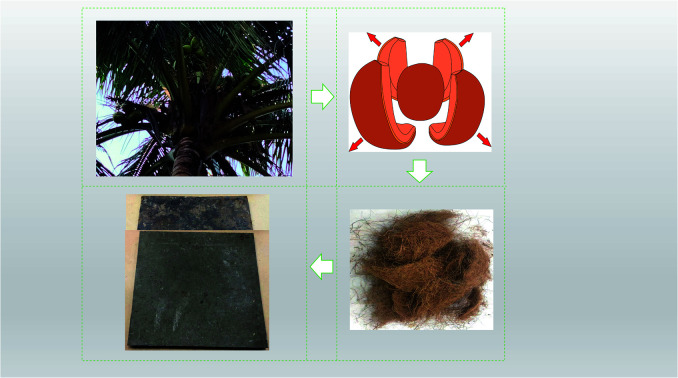

## 1. Introduction

Natural fiber-reinforced composite materials have received continuous attention due to their industrial application potential. Natural fibers are comparatively cheap, renewable, completely/partially recyclable, biodegradable, and eco-friendly,^1–6^ and synthetic products^7–12^ are continuously being replaced by natural products.^13–16^ The lignocellulosic fiber materials including flax, hemp, ramie, kenaf, jute, coir, hard and softwood materials, and rice husk are the biggest sources of biocomposite filler materials.^17,18^ Their availability, costing, lower density, and overall convenient mechanical features have made them attractive ecological materials as compared to synthetic fibers such as glass, carbon, nylon, and aramid. Natural fibers have a long history of usage for various products ranging from housing to construction and clothing.^19–22^ Natural fiber-reinforced composites are used in diverse applications such as automobiles, aerospace, construction and building sector, consumer products, packaging, and biomedicine. However, nowadays, synthetic fiber-reinforced products are still being used for producing composite materials because of the lack of adequate technology, research, and scientific innovations to utilize renewable natural fibers as a prominent replacement for biocomposite production.

Natural fibers are classified into different categories, such as animal, vegetable, and mineral fibers, and are further classified as seed, bast, stalk, grass/reeds, wood (hard and soft), and leaf fibers.^23,24^ Coir belongs to a popular seed fiber group; besides, as a lignocellulosic material, coir remains neutral in terms of CO_2_ emissions.^25,26^ Lignocellulosic materials are in line with the Kyoto protocol in terms of minimizing greenhouse gas emissions. However, there are some plants such as the banana plant, which are cultivated primarily for fruits; although, their leftover barks/leaves can be used as a potential biocomposite raw material.^27,28^ This fiber from banana is seldom used and is discarded just after collecting fruits. Fibers from coconut fruits also have a similar phenomenon just after collecting the fruits/coconuts water – they are discarded into the environment in general. Coconuts are grown in many parts of the world, especially in tropical and sub-tropical areas and play a significant role in economic development. It was reported that around fifty billion coconuts are produced throughout the world accumulating a huge quantity of coir fibers.^26,29^

Coconut husks are used for culinary purposes after extracting the copra and the interior liquid endosperm. The fruit shell of the coconut has a long decay time; hence, the transformation manufacturer and areas associated with high coconut consumption are facing challenges for disposing this waste through feasible and convenient disposal approaches.^30^ Another challenging aspect of coconut is that the husk and coconut fruits can float in ocean water without rotting for more than a month. Furthermore, durability is a major problem in natural fiber-reinforced composites; however, since coir fiber contains more lignin as compared to other natural fibers, it is more durable.^31^ Due to greater elongation at break properties, coir fiber-reinforced composites are also stretchable up to their elastic limit without rupturing.^31^ In this regard, fibers obtained from coconut husk are currently attracting attention from researchers and industrialists to determine more convenient routes for utilization.

The manufacturing approaches to natural fiber-reinforced composites are leaning toward novel and innovative routes for sustainable production. However, the biocomposite production from natural fiber reinforcement depends on various factors like interfacial fiber to matrix adhesions, length and contents of fiber, treatments of fibers, and the dispersions of polymers into the fiber structure. In this regard, researchers are becoming more interested in biocomposite manufacturing research^4,32–37^ and so coir fiber-reinforced composites^38–40^ are also getting significant consideration. Different researchers have reported promising results on developed coir fiber-reinforced biocomposites from different perspectives (thermal, mechanical, morphological, and so on). Rejeesh *et al.*^40^ have suggested that coir fiberboards could function as an alternative flame retardant material to other plywoods. Olveira *et al.*^41^ have proposed a design involving short coir fiber reinforced with epoxy thermosets through applying uniaxial pressure, characterized in terms of flexural properties, impact strength, and physical properties. The same study has further claimed that the perceived impact resistance and flexural modulus were satisfactory when 35% fiber volume with 375 g m^−2^ (fiber grammage/density) was used,^41^ although they found higher flexural strengths at 300 g m^−2^. Ayrilmis *et al.*^42^ reported coir fiber reinforcements with polypropylene (PP) in the presence of a coupling agent and found that the increased volume of the fiber loading negatively influenced the internal bonding strength and water resistance of the biocomposites. They also found an optimum fiber loading of coir (60%), up to which the tensile and flexural strengths of the composites increase.^42^

Natural fibers have very good compatibility with different thermoplastics, thermosetting polymers, or cementitious materials because of their lower density, better thermal insulation properties, mechanical properties, lower prices, unlimited availability, nontoxic-approaches, and problem-free disposals. Although the thermal, mechanical, and morphological properties of the natural fibers have been studied by so many researchers, the studies on coir fibers are still limited. Hence, this research reports various chemical, physical, morphological, and thermo-mechanical features of coir fiber-reinforced biocomposites. The potential application and economical features of coconut fiber-reinforced composites are further discussed and analyzed.

## 2. Coir fiber material

**Fig. 1:**
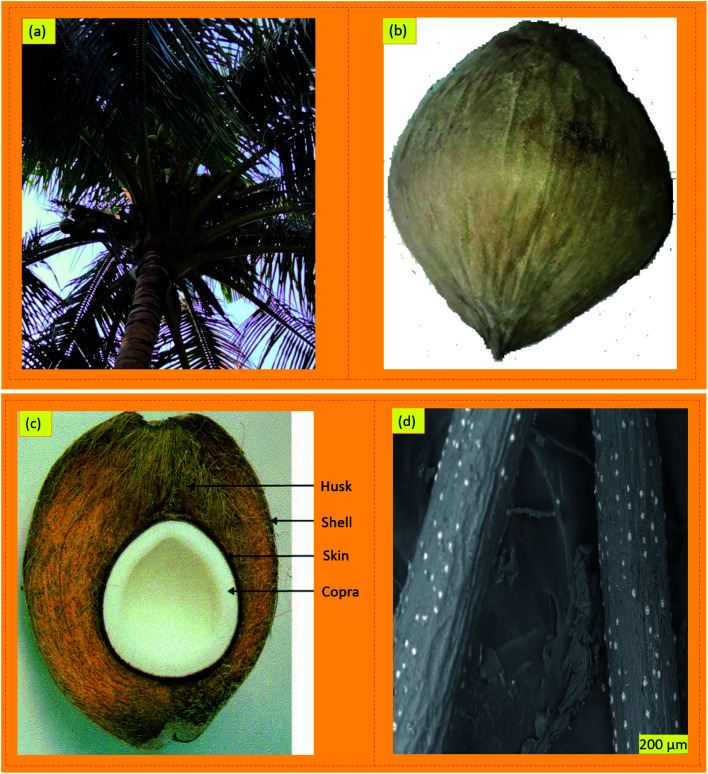
Photographs showing the physical and morphological structure of coconut plants and coir fiber: (a) coconut plants in Bangladesh (digital photographs taken by Muhammad Abu Taher); (b) coconut fruits (digital photographs taken by Muhammad Abu Taher); (c) cross-section of coconut fruits;^43^ (d) SEM image of coir fiber. Adapted with permission from Elsevier (c).^43^ Copyright, Elsevier 2004 (c).

A coconut tree can produce 50 to 100 coconut fruits per year.^44^ The photographs of the coconut palm tree, coconut fruits, coconut husk, and coir fiber morphology are provided in Fig. 1. The extracted fiber from the husks of the nut-shell is termed coir fiber. The fiber is extracted from the endocarp and external exocarp layers of coconut fruits. Generally, the extracted coir fibers are a golden or brown-reddish color just after removing and cleaning from coconut husks. The size of coir fiber threads is normally within 0.01 to 0.04 inches in diameter.^45^ Each coconut husk possesses 20 to 30% fibers of various fiber lengths.^46^ The coconut palm tree can also be considered an integral fiber-producing renewable resource due to the different parts of the palm like the petiole bark, leaf sheath, and leaf midrib.^47,48^ The majority of palm coconuts are produced in Indonesia, Sri Lanka, Brazil, the Philippines, Vietnam, Thailand, Malaysia, Bangladesh, and India.^49–52^ A study by Eldho *et al.* has mentioned that the coastal region of Asia produces 80% of the world's coconut fibers.^53^ The greater consumption of coconut fruits and water is generating green coconut trash, which is about 85% of the weight of the fruit. However, coir fibers are used as ropes, yarns, cords, floor furnishing materials, mattresses, sacking, brushes, insulation materials, geotextiles, and rugs. Coir fibers collected from coconut husks are thick and coarse, with some superior advantages like hard wearing capability, greater hardness quality (free from fragile characteristics like glass), better acoustic resistance, non-toxicity, moth-resistance, resistance to bacterial and fungal degradation, and they are not prone to exhibiting combustible properties.^42,54^ Besides, coir fibers have stronger resistance performances against moisture as compared to other plant-based natural fibers along with the ability to withstand salty water from the sea and heat exposure.^42^ The properties of mature coir fibers are as follows:

- 100% naturally originated fiber

- Coir fibers are strong and light

- Coir fibers easily withstand saline water

- Coir fibers easily withstand heat exposure

- Plastic shrinkage is delayed in coir-based materials by controlling the cracks developed at the initial stage

- The usage of coir in composite materials enhances thermal conductivity

- Biodegradability and renewability

- Higher water retention

- Rot-resistant

- Moth-resistant

- Heat insulator

- Have acoustic properties

**Fig. 2:**
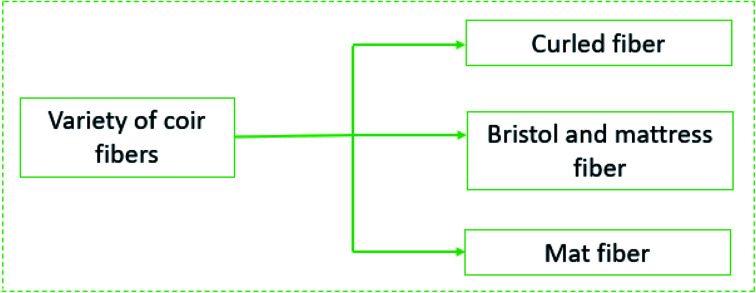
Different types of coir fibers.

Coir fibers can be of three types as shown in Fig. 2, namely, curled, bristol, and mat fibers.^45^ The curled fibers are of inferior quality and are short staple fibers. Bristol fibers are coarse and thick, obtained from extractions of dry coconut husks, and are also termed as brown fibers. Mat fiber is the best coir fiber type. It is obtained from retted coconut husks and has a longer and finer yarn. The mat fiber is highly resistant against bacterial attack.^45^

### 2.1 Retting of coir fibers

**Fig. 3:**
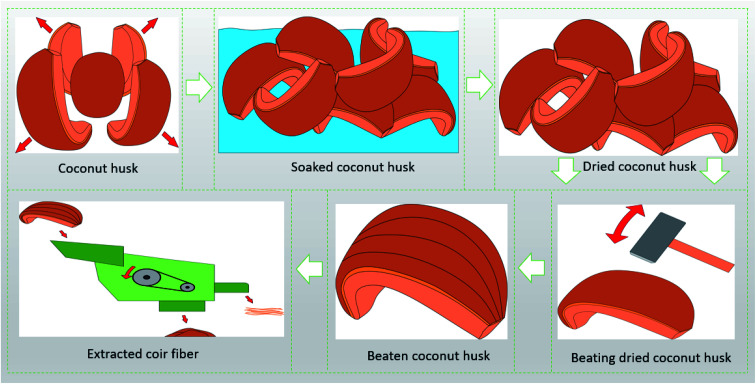
Proposed retting and extraction mechanisms of coir fibers from coconut fruits and husks.

Coir retting is performed in canals (a small area dug to store water), or rivers in riverine countries, or stored in watery areas; the coconut husks are submerged under the water by covering them with heavy soil. A mechanism regarding coir fiber retting is depicted in Fig. 3. Compared to other natural fibers like jute, coir fibers require longer times by at least 4 to 12 months for biological retting processes.^55,56^ The perfect retted coconut husks are separated from other poorly retted husks and washed with water to remove mud, sand, and slime from the surface. After that, the exocarp of the husk is easily peeled by hand. The coconut husks are then placed in a wooden box and beaten with wooden mallets or granite stones for further separation between the pith and coir fibers. Another washing cycle is carried out to further remove the surface impurities and the fibers are beaten again to ensure further separation of the pith and coir. Finally, the retted coir materials are sun-dried by spreading them over a mat. The fibers are then mechanically combed to process them for the next steps like spinning. The rotted husks could also be further mechanically processed for fiber extractions. The machine also softens and removes the piths entirely from fibers and provides parallel and clean fibers.^45^ The fibers required spinning are rolled in a roller for sliver formations. It was also found that tidal force is better than stagnant water for retting the coconut husks. The progression of the retting process results in the decrease/deterioration of pectin, fat, pentosan, and tannin contents but there is no loss of lignin or cellulosic substances.^45,57,58^ However, some of the researchers have also tried pollution- and hazard-free coir fiber treatment by using closed anaerobic reactor-based technology.^59^

### 2.2 Coir fiber extractions

**Fig. 4:**
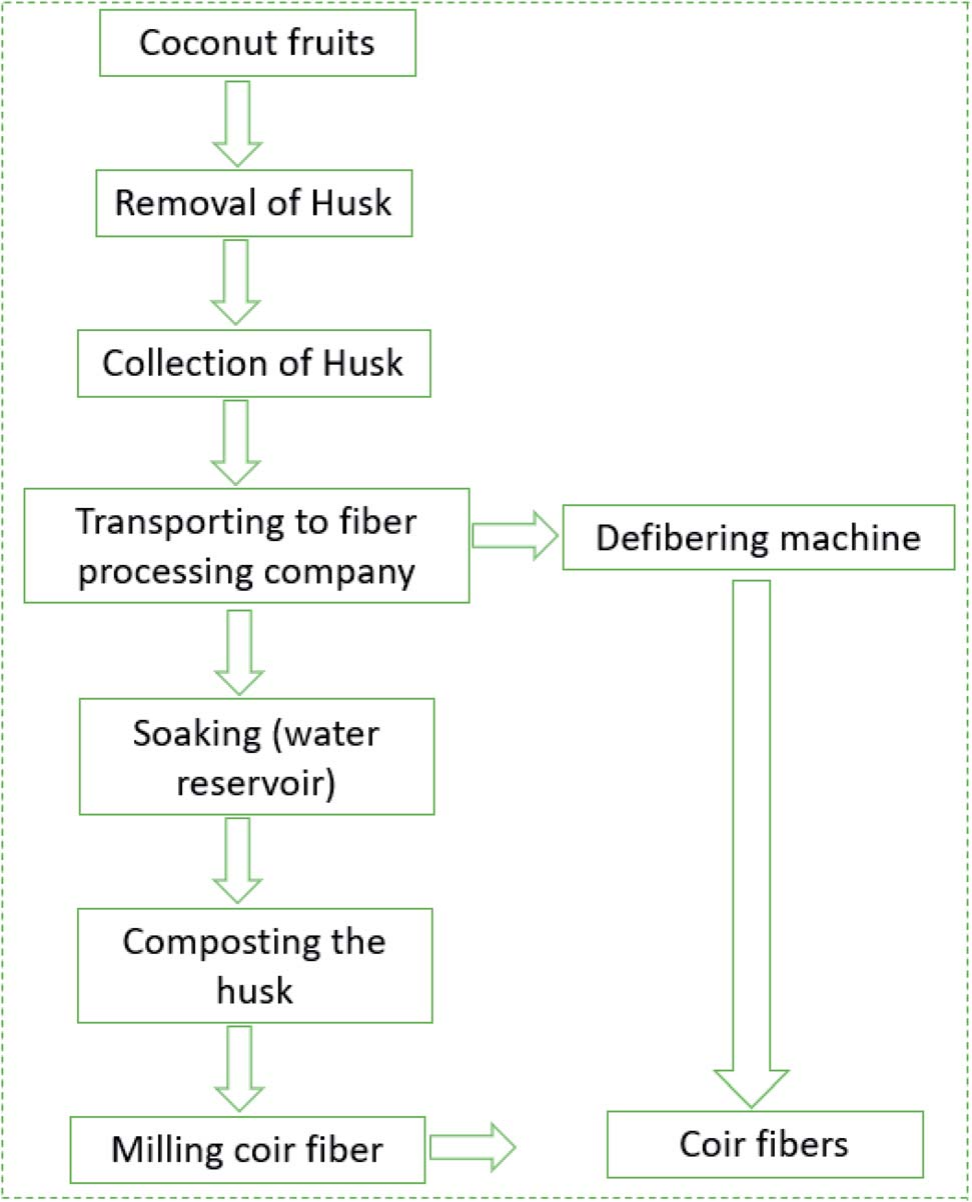
Coir fiber extraction flow process from coconut fruits.

There are several de-husking procedures available for the separation of coconut husks from the surface of fruits. A skilled farmer could manually split and peel around 2000 coconuts in a single day (approximately), whereas the household could do 1 to 2 coconuts per day, and hotels 10 to 20 coconuts in a day.^46^ An automatic de-husking machine could split and peel around 2000 coconuts every single hour.^46^ The coconut husks are collected by the fiber extraction industries from different sources that are not involved with direct de-husking operations (Fig. 3). The processes of fiber extractions are defined depending on the usage and quality of the fibers. Generally, the coconut husks in India are buried near the riverbanks in pits dug in a concrete tank filled with water. Sometimes, the coconut husks are also suspended through nets and weighted to ensure that they are submerged under the water in a river. Similar processes were described by Prashant *et al.*^46^ for processing coconut husks to extract coir materials. A schematic flow process and fiber extraction method is shown in Fig. 3 and 4.

### 2.3 Coir-based nanocellulose

Nanotechnology has become a hot topic nowadays, especially for nanocomposites developed through extracting nanocellulose from different natural fiber-based materials.^60–64^ The cellulose fibrils can be easily cleaved when hydrolyzed with acidic solutions in small particles, which are termed micro-crystalline cellulose, nanocellulose, cellulose nanowhiskers, and cellulose nanocrystals.^65^ Nanocrystalline cellulose has certain benefits as compared to other nano-structured materials.^65^ The extraction of nanocellulose from coir husk could be another prominent raw material for nanocomposite production. Generally, coir fiber-based manufacturing industries use the coir materials just after the extraction without any additional processing. However, the nanotechnology-based functionalization or treatment of coir materials needs satisfactory and feasible extraction protocols. The separation of nanocellulose from coconut husk could open another new door for industrially advanced composite materials. There are several pretreatment methods used for isolating nanocellulose fibers from coconut. Steam explosion is one of the most attractive and popular technologies in this regard.^53^ Machado *et al.*^65^ reported a plasticized nanocomposite developed from biodegradable cassava starch film with glycerol and coir fiber-derived nanocellulose (length/diameter value 38.9 ± 4.7 after acidic hydrolysis, performed at 50 °C for 10–15 min in the presence of 64% H_2_SO_4_). They further found that the as-produced composites provided higher tensile modulus but there was a decline in the elongation modulus.^65^

### 2.4 Coir fiber compositions

The composition of fiber depends on the types of extracted plants and agricultural conditions.^66,67^ Generally, cellulose, lignin, and hemicelluloses are three chemical constituents of plant-based fibers, whereas the cellulose and hemicelluloses are polysaccharides and lignin is a three-dimensional (3D) amorphous polyphenolic macromolecule, comprised of three different types of phenylypropane units.^68,69^ The celluloses are crystalline, whereas lignin is amorphous.^70^ However, the lignin is normally located at the fiber surface, whereas the cellulose acts as the backbone of the natural fibers. The coir fibers are composed of cellulose, lignin, hemicellulose, pectin, ash, and other water-soluble elements as shown in Table 1. It was found that coir fibers have approximately 40 to 50% lignin, 27 to 45% cellulose, 0.15 to 20% hemicellulose, 3.5% ash, and 9 to11% moisture content (Table 1). In contrast to other natural fibers, coir fibers contain more lignin but less cellulosic polymers.^71^ However, the higher lignin contents of coir make it harder and naturally rigid. Besides, the resiliency, rot and damp-resistance properties and water absorption capability have made it exceptionally convenient for multifaceted applications. Coir also provides wonderful hard-wearing and endurance features along with weather resistance characteristics, which make it suitable for cords, brushes, and rope-based applications. The enriched lignin and cellulose contents of coir have made it an excellent candidate for biocomposite production as compared to other natural fibers as a potential filler material due to its inherent properties like strength and modulus.^72^ The higher lignin but relatively lower cellulose content of coir results in elongation at break as well as the tensile strength of coir fiber-reinforced composites.

**Table 1:** Chemical properties of coir and different natural fibers

Fiber and sources	Cellulose	Lignin	Hemicellulose	Pectin/wax	Ash	Moisture content	Ref.
Coir (Zainudin *et al.*)	32–43	40–45	0.15–0.25	—	—	—	73
Coir (Narendar *et al.*)	27.41	42.0	14.63	10.16	—	—	74
Coir (Verma *et al.*)	37	42	—	—	—	—	71
Coir (Malkapuram *et al.*)	36–43	41–45	10–20	3–4	—	—	75
Coir (Barbosa Jr *et al.*)	43.4 ± 1.2	48.3 ± 1.9	4.0 ± 0.03	—	3.5 ± 0.2	10.2 ± 0.2	76
Coir (Abraham *et al.*)	39.3 (±4)	49.2 (±5)	2 (±0.5)	—	—	9.8 ± 0.5	53
Flax (Kabir *et al.*)	71	2.2	18.6–20.6	2.3/1.7		10.0	77
Kapok (Raju *et al.*)	35	21	32	—	—	—	78
Bamboo (Hasan *et al.*)	73.83	10.15	12.49	0.37		3.16–8.9	1
Sugarcane bagasse (Raju *et al.*)	55.2	25.3	16.8	—	—	—	78
Jute (Kabir *et al.*)	67–71.5	12–13	13.6–20.4	0.2/0.5	—	12.6	77
Hemp (Kabir *et al.*)	70.2–74.4	3.7–5.7	17.9–22.4	0.9/0.8	—	10.8	77
Ramie (Kabir *et al.*)	68.8–76.2	0.7–0.6	13.1–16.7	1.9/0.3	—	8.0	77
Sisal (Kabir *et al.*)	67–68	8.0–11.0	10.0–14.2	10.0/2.0	—	11.0	77
Pineapple (Raju *et al.*)	82	12	—	—	—	—	78

### 2.5 Structural properties of coir fiber

**Fig. 5:**
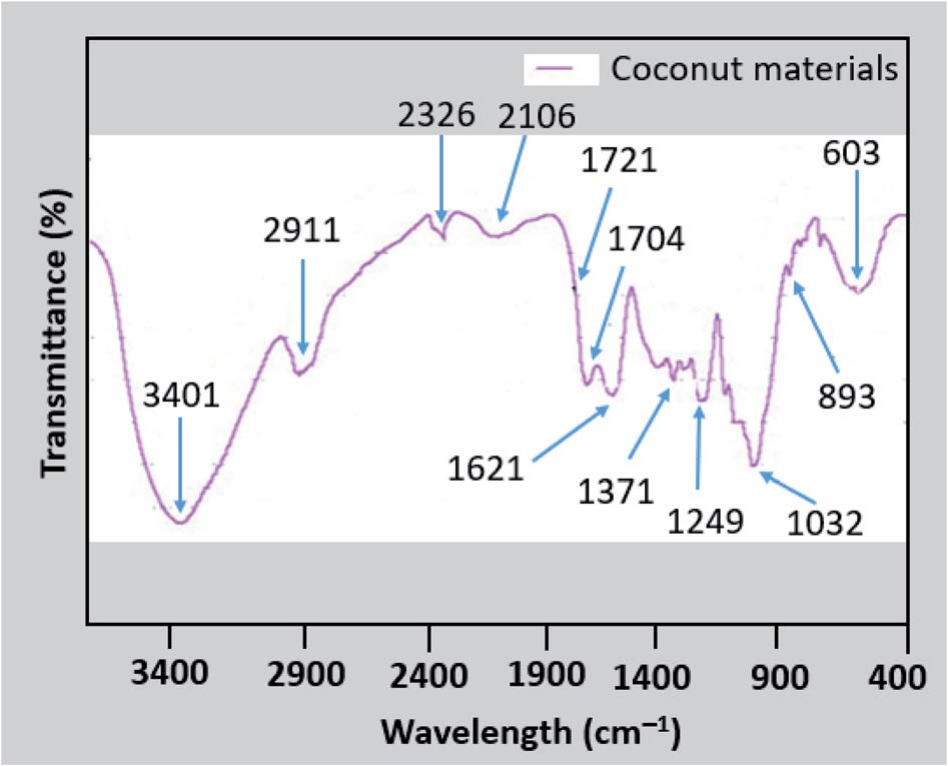
FTIR analysis of coconut materials. Copyright, Elsevier 2010. Adapted with permission from Elsevier, 2010.^79^

A typical FTIR analysis (spectra and associated peaks in tabulated form) of coir and other natural fibers is shown in Fig. 5 and Tables 2 and 3. The peak at 3401 cm^−1^ is associated with O–H stretching vibrations, which is a typical characteristic of natural fibers (Table 3).^2,79^ The broad absorption peak is associated with the hydrophilic characteristics of the coconut materials, indicating the presence of the –OH group in aromatic and aliphatic alcohols. The peak at 2911 cm^−1^ is responsible for the symmetric and asymmetric stretching of C–H, which is related to the methylene and methyl groups. The aliphatic moieties of hemicellulose and cellulose are indicated by these two stretching peaks.^80,81^ The absorption band at 1721 cm^−1^ is related to the stretching of C=O groups in the uronic ester and acetyl groups or carboxylic group of coumaric and ferulic acids of lignin.^81,82^ The presence of amide I is reflected by the peak at 1621 cm^−1^. The vibration frequency depends on the hydrogen bonding nature of N–H and C=O groups and protein secondary structures.^80,81^ The deformation of C–O is related to the peaks at 1030 and 1086 cm^−1^. The overall FTIR study shows the significant presence of the chemical constituents of coir materials. Some other relevant information on FTIR studies on coir materials is tabulated in Table 2.

**Table 2:** FTIR analysis of coconut materials. Copyright, Elsevier 2010. Adapted with permission from Elsevier, 2010.^79^

Location of peaks (cm^−1^)	Assignment	Coconut materials
3460–3400	Stretching of O–H	3401
3000–2850	C–H symmetric and asymmetric stretching related to methylene and methyl groups	2911
2400–2300	Stretching vibrations of P–H and P–O–H	2326
2200–2100	Stretching of Si–H	2101
1738–1700	Stretching of C=O in uronic ester and acetyl group or carboxylic group of coumaric and ferulic acids	1721
1650–1580	Bending of N–H in primary amines	1621
1375–1350	Stretching of C–H in phenolic and methyl alcohol or rocking of C–H in alkanes	1371
1250–1200	Stretching of Si–CH_2_ in alkanes or C–O plus C–C plus C=O	1249
1086–1030	Deformation of C–O in secondary alcohol and aromatic or aliphatic C–H in plan deformation plus deformations of C–O in primary alcohol	1032
900–875	Frequency of C-1 group/ring	893

**Table 3:** Typical FTIR analysis of different natural fibers.^83–87^

Stretching/bonding	Jute (cm^−1^)	Hemp (cm^−1^)	Kenaf (cm^−1^)	Kapok (cm^−1^)	Sisal (cm^−1^)	Pineapple leaf (cm^−1^)
C–H	1255.6	—	—	1245.5	1259.9	—
C–H	1383.1	1384.1	—	1383.6	1384.1	1374.2
C=C	1596.1	1654	—	1596.1	1653.9	1608.3
C=O	1741.1	—	1736	1741.1	1736.5	1737.4
C–H	2918.1	2920.5	2899	2918.1	2924.2	2903.8
–OH	3419.7	3448	3338	3419.7	3447.2	3349.9

### 2.6 Physical and mechanical properties of coir fibers

The ultimate mechanical properties of the coir fiber-reinforced biocomposites are also significantly influenced by the characteristics of the control coir materials.^71,88^ In this regard, it is necessary to study the chemical and physical characteristics of coir materials before the fabrication of biocomposites. Some of the recently reported chemical and physical properties are tabulated in Tables 1 and 4 for coir materials and some other commonly used natural fibers. The most significant physical properties of the coir fibers include density, strength, elastic modulus, and elongation at break, whereas the chemical characteristics are variable in terms of lignin, cellulose, and hemicelluloses. It could be concluded that coir fibers have a density of around 1.15 to 1.45 g cm^−3^, an elastic modulus of 4 to 7 GPa, 54 to 250 MPa strength, and 3 to 40% elongation at break (%), depending on the type, origin, nature, and processing of the fiber (Table 4). The different concentrations of lignin contained in coir also influence the variable mechanical properties as shown in Table 5.

**Table 4:** Mechanical properties of coir and different commonly used natural fibers

Sources	Elastic modulus (GPa)	Strength (MPa)	Density (g cm^−3^)	Elongation at break (%)	Ref.
Coir (Tran *et al.*)	4.6–4.9	210–250	1.3	—	89
Coir (Malkpuram *et al.*)	4–6	131–175	1.15	15–40	75
Coir (Defoirdt *et al.* and Nam *et al.*)	4–7	186–345	1.29	—	90 and 91
Coir (Balaji *et al.*)	—	54	1.45	3–7	92
Coir (Barbosa Jr *et al.*)	—	120 ± 5	—	8.0 ± 1.0	76
Flax (Kabir *et al.*)	30–60	345–1100	1.5	0.2–0.7	77
Abaca (Mahmud *et al.*)	12	430–760	1.5	3–10	18
Bamboo (Hasan *et al.*)	27–40	500–575	1.2–1.5	1.9–3.2	1
Sugarcane bagasse (Hasan *et al.*)	5.1–6.2	170–350	1.1–1.6	6.3–7.9	1
Jute (Kabir *et al.*)	13–26.5	393–793	1.3–1.4	1.16–1.5	77
Hemp (Kabir *et al.*)	30–60	690	1.5	1.6	77
Ramie (Kabir *et al.*)	61.4–128	400–938	1.5	1.2–3.8	77
Sisal (Kabir *et al.*)	9.4–22.0	468–640	1.45	3–7	77
Pineapple (Pai *et al.*)	34.5–82.5	413–1627	1.52–1.56	—	93

**Table 5:** Effects of lignin content on the mechanical properties of coir fiber. Adapted with permission from Elsevier, 2011 ^68^

Coconut fiber	Tensile strength (MPa)	Young's modulus (GPa)	Elongation at break (%)
L 42 fiber	123.2 ± 34.7	2.29 ± 0.47	33.39 ± 7.01
L 31 fiber	97.3 ± 37.4	2.59 ± 0.64	21.61 ± 9.00
L 21 fiber	112.5 ± 47.8	2.43 ± 0.62	27.59 ± 11.95

### 2.7 Treatment of coir fibers

**Fig. 6:**
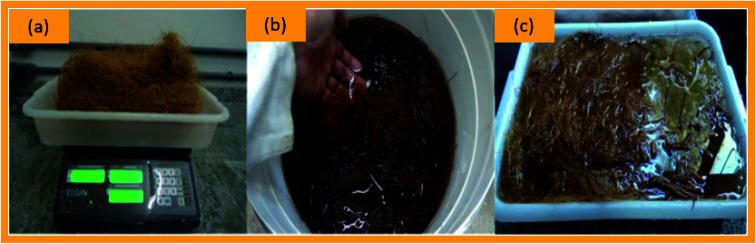
Treatment of coir fiber materials: (a) control coir fiber, (b) coir fiber in Na_2_CO_3_ solution bath, and (c) post-treatment washing of coir fiber. Adapted with permission from Elsevier.^96^ Copyright, Elsevier 2010.

The interfacial adhesion characteristics between the natural fiber and matrix is an extremely important parameter that significantly affects the mechanical features of biocomposites through enabling stress transfer from the polymeric matrix to fibers.^94^ The chemical cross-linking or physical origination could impact the adhesion of the fibers and polymers in the biocomposites. Besides, the chemical bonding could also significantly affect the biocomposite interface quality. As a polyphenolic element, lignin plays a major role in natural fiber/matrix adhesions. Mir *et al.*^95^ has reported that the treatment of coir fiber in a single-stage by Cr_2_(SO_4_)_3_·12H_2_O and double-stage by NaHCO_3_ and CrSO_4_ caused an increase in Young's modulus but a decrease in the tensile strength in terms of the increased span lengths of fiber. However, the same study^95^ further found that the treated coir fibers provided higher tensile strengths as compared to untreated coir materials. Muensri *et al.* found an interesting effect on sodium chlorite treated coir fibers, namely, a reduction in the lignin content from 42 to 21 wt% after the treatment.^68^ A proposed treatment process of coir is depicted in Fig. 6. The surface treatments of coir fibers are bleaching, mercerization, dewaxing, acetylation, acrylation, cyanoethylation, benzoylation, silane treatment, stem explosion, isocyanate treatments, and so on. Some commonly implemented treatment processes are outlined in this section.

#### 2.7.1 Mercerization or alkali treatment

This is the most commonly used and popular method for natural fiber pretreatment to modify the surface. A disrupted hydrogen bond is created with the natural fibers with enhanced surface roughness.^97^ Different surface impurities like oil, wax, and fats are removed from the cell membranes of the fiber due to alkaline treatments. Alkaline reagents like NaOH aqueous solutions assist the natural fibers to ionize –OH groups into the alkoxide.^98^ The degree of polymerization, molecular orientation, and chemical composition are affected by the alkaline treatments, which impact the mechanical performances of the treated fiber-based composites. A proposed reaction mechanism is shown in eqn (1).

1 Coconut materials–OH + NaOH → coconut materials–O–Na + H_2_O

#### 2.7.2 Silane treatment

The treatment of coir fibers with silane reduces the –OH groups and enhances the surface interface. Silane coupling agents enhance the crosslinking in the interface area.^98^ Silane functions perfectly to improve the interface between the natural fibers and the associated matrix. Consequently, the mechanical features of the biocomposites are also improved. Javadi *et al.*^99^ researched the silane treatment of coir fibers, where a 2% concentration of silane (on the weight of coir) was used. They used a K-mixer instrument, where they operated the machine at 5000 rpm at 150 °C.^99^ The silane treatment could reduce the water absorption characteristics of natural fiber-reinforced composites.^100^ This mixer ensured the uniform dispersion of silane on coir fibers. A silane treatment reaction mechanism^98^ is shown in eqn (2) and (3).

2 CH_2_CHSi(OC_2_H_5_)_3_ → CH_2_CHSi(OH)_3_ + 3C_2_H_5_OH

3 CH_2_CHSi(OH)_3_ + coir–OH → CH_2_CHSi(OH)_2_O–coir + H_2_O

#### 2.7.3 Maleated coupling agents

The biocomposites are strengthened by using maleated coupling agents with natural fibers and the associated matrix. Besides, the interfacial bonding of the fiber and matrix is improved by using maleated coupling agents. Ayrilmis *et al.*^101^ developed a composite panel for automotive applications (interior) by using maleic anhydride-grafted polypropylene (PP) or MAPP with different loadings of coir and found an optimum recipe (3 wt% MAPP, 37 wt% PP, and 60 wt% coir fiber).

#### 2.7.4 Acetylation

The acetylation approach for treating the natural fibers is also termed the esterification method to plasticize the cellulosic materials.^102^ The natural fiber acetylation is performed through grafting acetyl groups with the cellulosic structures of fibers.^102^ A proposed reaction mechanism is shown in eqn (4).

4 Coir–OH + CH_3_CO–OH → coir–OCOCH_3_

#### 2.7.5 Benzoylation treatments

The hydrophilic nature of natural fiber, as well as coir fibers, creates adhesion problems with hydrophobic polymeric materials; the benzoylation treatment of natural fibers could address this challenge to increase mechanical properties. The thermal stability of the coir fiber could further be improved by using this method.^103,104^ In this regard, alkaline treatment is initially carried out on the coir fiber surface to ensure that –OH groups are exposed on the surface. Benzoyl chloride treatment is then conducted on the fiber, which in turn replaces the –OH group and strongly attaches to the backbone of cellulose. The above-mentioned circumstances improve the hydrophobicity of fibers, thus increasing the fiber-to-polymer adhesions.^105^

## 3. Polymers used for coir fiber-reinforced composites

Coir fibers show tremendous potential for reinforcements with thermoplastic,^38,106–111^ thermosetting,^112–119^ and cementitious matrixes.^120–125^ Thermoplastic polymers like polylactic acid (PLA), PP, polyethylene (PE) and high-density polyethylene (HDPE) are widely used for producing coir fiber-reinforced biocomposites. The incorporation of thermoplastic polymers into coir enhances the thermomechanical properties of the biocomposite. The waxy layer of coir fiber makes strong bonds with thermoplastic polymers, thus increasing the strength.^126^ The use of thermosetting polymers like PES (polyester), MUF (melamine-urea-formaldehyde), epoxy resin, *etc.* is another promising area of research for coir fiber-reinforced biocomposites. Biswas *et al.*^127^ mentioned that the pretreatment of coir fibers could provide better mechanical performances to the coir fiber-reinforced thermosetting polymeric matrix. The pretreatment of coir ensures greater adhesion between the fiber and polymeric matrix since normally (without treatment), hydrophilic fibers restrict efficient adhesion with the polymers.^127^ The biodegradability property of the composites made from coir/epoxy is enhanced after the pretreatment, as reported by another study.^114^ The cementitious matrix from coir and cement also shows great potential in developing composite panels for building and construction. Since the coir fibers contain some outstanding features as an emerging natural fiber, the manufacturing of light-weight cementitious matrix has gained popularity from coir fiber-reinforced cement composites. The availability of raw materials and cheaper costs are some of the key features for the products of the construction and building sector, hence coir fiber shows a new milestone in this perspective. Abraham *et al.* developed green building materials from optimized volumes of coir (10%), which provided satisfactory performance characteristics as roofing tiles.^128^ The mechanical and physical properties of different coir fiber-reinforced composites are tabulated in Table 6. According to the results, it could be summarized that coir fiber-reinforced composite materials are going to dominate the composite sectors in the near future.

**Table 6:** Mechanical properties of coir and different natural fiber-reinforced composite materials^a^

Biocomposite materials	*ρ* (kg m^−3^)	TS (MPa)	MOR (MPa)	TM (GPa)	IBS (MPa)	IS (kJ m^−2^)	ThS (%)	WA (%)	Ref.
Coir/PP	749 (10)	13.2 (0.49)	24.3 (0.8)	2.54 (0.079)	1.89 (0.18)	—	3.94 (0.2)	10.26 (0.59)	101
Coir/PP	—	42.5 ± 0.7	52.±2	2.17	—	—	—	—	152
Coir/PLA	—	57.9 ± 0.6	107.1 ± 1.4	4.2 ± 0.3	—	—	—	—	134
Coir/PLA	—	30.7 ± 0.7	101.5 ± 1.6	4.9 ± 0.5	—	15.1 ± 0.4	—	—	153
Coir/epoxy	—	17.9	40.09	2.59	—	6.07	—	—	96
Coir/PES	—	18.56	24.19	—	—	48.02	—	—	154
Coir/epoxy	—	5.22 ± 0.3	32.87 ± 0.3	—	—	101.35 ± 0.4	—	—	155
Coir/cement	1450	—	5.01	—	—	—	—	30	156
Coir/cement	—	—	2.6	1.04	0.26	—	0.79	30.66	157
Coir/PES	—	14.86	39.12	—	—	124.23	—	—	158
Coir/epoxy	—	13.05	35.42	—	—	17.5	—	—	127
Flax (woven-warp direction)/bioepoxy	—	84.66	116.53	6.39	—	—	—	—	159
Abaca/PP	—	40–50	70–80	—	—	4–4.5	—	—	160
Agave/PP	—	282 ± 9.34	—	8.4 ± 2.67	—	—	—	—	161
Sugarcane bagasse/cement	1596	—	2.9	—	—	30	0.38	6.00	162
Jute (non-woven)/PLA	—	55 ± 11.5	67 ± 8.4	0.87 ± 0.02	—	12.98 ± 1.1	—	—	163
Hemp/thermoplastic polyurethane	—	24.18 ± 6.55	19.5 ± 0.91	0.537 ± 0.059	—	—	—	—	164
Ramie	—	54.88	99.78	9.13	—	—	—	—	165
Sisal/benzoxazine/epoxy	—	64	75	1.4	—	22.4	—	—	166
Pineapple leaf fiber/PP	—	61	31	1.096	—	4.61	—	—	167

a *ρ* – density; TS – tensile strength; MOR – modulus of rupture; TM – tensile modulus; IBS – internal bonding strength; IS – impact strength; ThS – thickness swelling; WA – water absorbency.

## 4. Fabrication of coir fiber-reinforced composites

Fabrication is a very important aspect that requires focus for biocomposite manufacturing. Different manufacturing methods are used for coir fiber-reinforced composites. The compression, extrusion, injection molding, RTM (resin transfer molding), and open molding methods are some of the popular fabrication techniques for coir fiber-reinforced composites. However, some processing parameters (like fiber volume, type of fiber, temperature, pressure, moisture content, *etc.*) need to be considered during biocomposite manufacturing to produce successful products. Different fabrication methods are described in this section.

### 4.1 Compression molding

Compression molding is considered as the most suitable method for producing high-volume composite parts, both from thermoplastic or thermosetting polymers, or even cementitious materials.^2,3,129,130^ Whether the fiber length is long or short, both could be processed using the compression molding technique. It is nearly the same approach as the hand lay-up process, except that the matching dies used are closed during applying the pressure at a certain temperature for perfect curing. This method is more appropriate if the dimension of composite is smaller; however, open molding or hand layup is more feasible in the case of larger composite panels. Compression molding could be implemented in two different ways^131^ as indicated below:

• Cold compression: operation is performed at room temperature without using any temperature on the mold.

• Hot compression: the operation is carried out in terms of certain temperatures and pressures on the mold.

The high-quality composite panels could be manufactured by using this method through controlling and regulating some key parameters like temperature, pressure, and time. Besides, the physical dimensions of the composite panels like length, width, and thickness of the composites need to be selected carefully along with associated materials to be used for manufacturing the composites.

### 4.2 Extrusion molding

A screw extruder is used for this molding process at a specific speed and temperature. The composite materials need to cool down when the extrusion process is complete and could be molded further as per the desired specifications. Extrusion molding is used for thermoplastic polymer reinforced composites with improved mechanical strength and stiffness.^132^ Different studies have been conducted for coir fiber reinforcements with the extrusion molding process.^133–136^

### 4.3 Injection molding

Injection molding facilitates diversified processing feasibility for polymeric composite manufacturing, especially for high-volume production. With shorter cycle time along with post-post-processing operation/functioning, the injection molding provides exceptional dimensional stability to the biocomposite materials. However, some limitations remain for using injection molding methods; *e.g.*, it requires the lower molecular weight of polymers for maintaining adequate viscosity. Besides, the length of fiber and processing temperatures also have less influence on the produced biocomposite performance.^137–139^ It has also been reported that plant fiber reinforced with PP composites displayed higher performances in the case of injection molding as compared to the compression molding techniques.^139,140^

### 4.4 RTM method

The RTM method provides high-quality finishing on composite surfaces with better dimensional accuracy. The thermoset polymeric resins are transferred to a closed mold at low temperature and pressure. Fibers of different forms could function as reinforcements by applying RTM methods. Although RTM is advantageous in terms of the ecological, economical, and technological perspectives, some factors also need consideration, such as fiber concentrations, edge flow, and fiber washing.^141^ However, the most prominent advantage of using RTM methods for natural fiber reinforcement is the positive contribution towards the strength and stiffness of the biocomposites.^142,143^

### 4.5 Open molding

Thermoset polymer-reinforced composites with natural fibers are manufactured by using this method. The biocomposites are cured at ambient temperature in an open mold where the natural mold (fibers as reinforcement materials and thermoset as matrix materials) are placed. The investment in equipment is not high for producing high-volume thermoset polymeric composites by using this technology, although this method also has some critical drawbacks like longer curation time, manual labor, and higher waste generations with non-uniform products.^31^ Through implementing spraying up/hand layup, the open molding process could be designed. In this regard, the open molding method is also considered the most economical method for biocomposite products.

## 5. Properties of coir fiber-reinforced composites

Tensile, flexural, and impact properties are some of the significant mechanical properties of natural fiber as well as coir fiber-reinforced composites. The mechanical and physical properties of different coir and natural fiber-based composites are tabulated (Table 6). It was found that coir fibers provide significant tensile, flexural, impact, water absorption, and thickness swelling properties from developed biocomposites. However, different factors affect the mechanical performances of coir fiber-reinforced composites as given below:

- Types of coir fiber

- Geometry of coir fiber

- Processing of coir fiber

- Orientation of coir fiber

- Surface modification of coir fiber, and

- Fabrication of coir fiber

### 5.1 Tensile properties

Tensile properties are mainly influenced by the interfacial adhesion characteristics between the coir and matrix polymer. Coir has greater proportions of lignin than other natural fibers, which facilitates greater tensile strengths.^95^ Siddika *et al.*^144^ determined the tensile strength of coir fiber-reinforced PP composites as per ASTM D 638-01 standard by using a universal testing machine with 4 mm min^−1^ crosshead movement. They conducted the test until the failure of the test samples. Romli *et al.*^145^ researched the factorial design of coir-reinforced epoxy composites to investigate the effects of compression load, fiber volume, and curation time and found that fiber volume has the most significant influence on the produced composites (tested *via* ANOVA in terms of tensile strength).

### 5.2 Flexural properties

The flexural strength of biocomposites indicates their resistance to bending deformations. The modulus of biocomposites and associated moments of inertia are two main dependent parameters of flexural properties.^146^ However, it is necessary to ensure an optimum loading of coir fiber to achieve the required flexural properties. Ferraz *et al.*^147^ conducted a study on differently-treated coir fiber-reinforced cementitious composites, where they found that hot water treatment provided an increase in the MOE (modulus of elasticity) but alkaline treatment caused a decline in the mechanical and physical properties of coir/cement composite panels. In another study by Prasad *et al.*,^148^ it was reported that flexural strengths started to decline after 20% coir fiber loading, whereas it increased up to 20% fiber loading (providing highest bending strength by 141.042 MPa). This test was conducted as per ASTM D 7264 on different coir fiber loadings on polyester thermoset resins.^148^ Siddika *et al.*^144^ conducted a flexural study according to the standard ASTM D 790-00 to assess the bending properties of biocomposites developed from coir. Coir fiber reinforced with magnesium phosphate reinforced composites provided higher flexural strengths with increased fiber loading up to an optimum level then it declined again.^149^

### 5.3 Impact strength

The Charpy impact strength testing equipment is used for impact strength measurements. The brittle and ductile transition of biocomposites could also be investigated by using this method. The level of bonding between the natural fibers and matrix is responsible for the impact strengths of natural fiber-reinforced composites.^146^ The parameters such as the composition of natural fibers like the toughness of polymers, surface treatments, and interfacial bonding between fiber and matrix could enhance the biocomposites' tensile and flexural performances but decline the impact strengths.^150^ However, the serviceability of the natural fiber-reinforced composite is dependent on the impact strength of natural fibers.^146^ Siddika *et al.*^144^ performed the impact strength characterization by using a Charpy impact tester (MT3116) as per ASTM D 6110-97. The same study has further claimed that with the increased fiber loading, more force is required for pulling-out the fibers, hence the impact strength increases.^144^ Padmaraj *et al.*^151^ reported that alkali-treated coir fiber-reinforced unsaturated polyester composites provided 22.2 kJ m^−2^ impact strength.

### 5.4 Coir fiber-reinforced hybrid composites

Typically, hybrid composites are manufactured by reinforcing two or more different types of fiber materials along with a common polymeric matrix.^168^ Generally, hybrid composites reinforced with different natural fibers demonstrate greater mechanical performances as compared to single-fiber-reinforced composites, which are even competitive with synthetic fiber-reinforced composites if the fibers are carefully selected as per the requirements.^169^ In the case of hybrid composites, the volume fraction of the associated fibers strongly influences the mechanical performances of the composites and stress transfer between the reinforcements (fiber) and polymers in the matrix system.^170^ Reinforcing synthetic fibers with natural fibers is also becoming a popular hybridization technology for developed hybrid composites. The natural fibers show significant potential in terms of replacing synthetic fibers for developing hybrid composites having superior mechanical and functional properties through minimizing material and production costs. Tran *et al.*^89^ reported that the reinforcement of bamboo with coir fiber could positively influence the failure at strain, hence the incorporated bamboo fiber materials could enhance the stiffness of coir fiber-reinforced polymeric composites (Table 7).

**Table 7:** Mechanical properties of hybrid composites, through reinforcing coir fibers with different natural fibers^a^

Hybrid composites	TS (MPa)	TM (GPa)	MOR (MPa)	FM (GPa)	IS (kJ m^−2^)	EB (%)	Ref.
Coir/silk/polyester resin	15.62	43.74	—	—	—		168
Coir (75%)/jute (25%)/PP	13.46 ± 0.39	1.03 ± 0.11	16.48 ± 3.24	0.90 ± 0.18	0.387 ± 0.004		171
Coir/bamboo/PP	87.6 ± 4.4	7.3 ± 0.9	—	—	—	2.2 ± 0.8	89
Coir/glass/polyester	29.17	0.98	73.04	64.23	64.23	8.85	172
Banana stem (10%)/coir (10%)/MAPP	36.2 ± 2.6	1.09 ± 0.0096	32.6 ± 3.4	—	9.3 ± 1.1		173
Coir (22.5%)/sugarcane leaf sheath (7.5%)/PES	13.42	1.04	25.84	2.17	—		174
Coir(90%)/pineapple (10%)/epoxy	43.53	29.41	16.09	28.57	—	85.54	175
Coir pith/nylon/epoxy	7.57 ± 0.3	—	53.19 ± 0.4	—	—		176
Coir/date palm/epoxy	46.75	7.54	—	—	—	0.62	177
Coir (20 g)/luffa (5.7 g)/epoxy	51.32	39.4	—	—	43.21		178
Coir (30%)/carbon fiber/epoxy	285.74		215.79				179
Coir (15%)/agave (15%)/epoxy	48.37	0.33	80.53	4.98			180

a TS – tensile strength; TM – tensile modulus; MOR – modulus of rupture; FM – flexural modulus; IS – impact strength; EB – elongation at break, MAPP – maleic anhydride grafted polypropylene.

### 5.5 Morphological properties

**Fig. 7:**
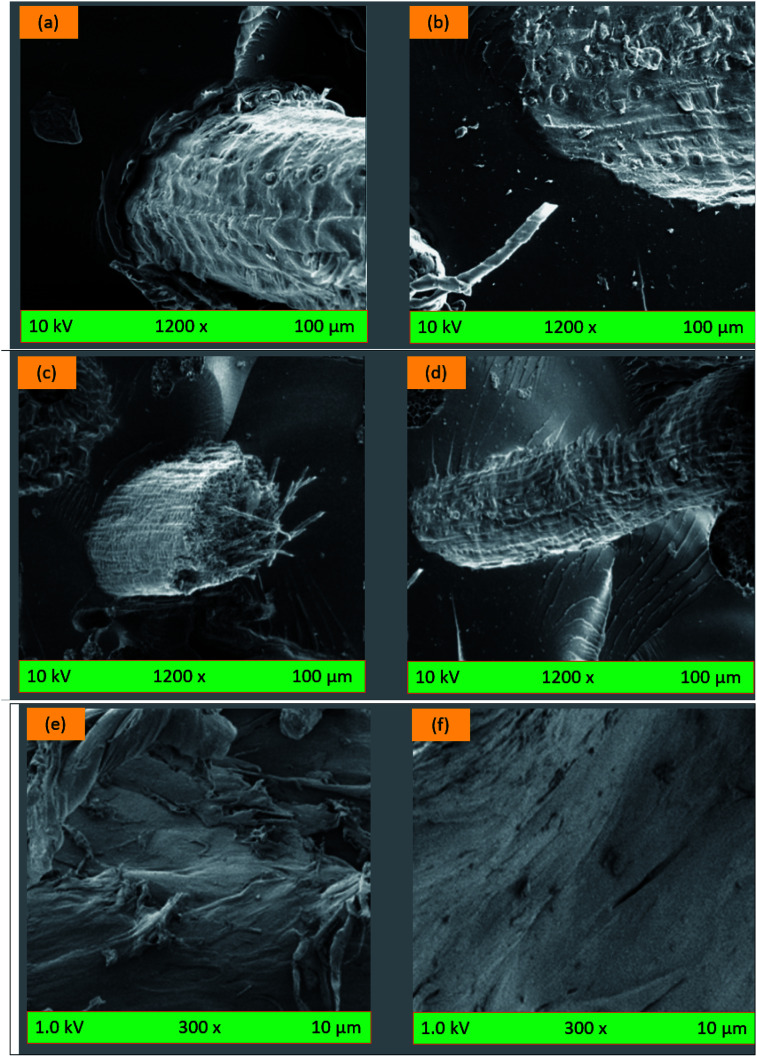
SEM photographs of coir fiber/epoxy biocomposites (a–d): (a) before treatment, (b) after treatment, (c) fractured composites before treatment, (d) fractured composites after treatment. (e) Untreated coir/PP composites, and (f) treated coir/PP composites. Adapted with permission from Elsevier.^182,184^ Copyright, Elsevier 2016 and 2010.

The effects of adhesion properties on coir fiber-reinforced composites were easily observed through the SEM (scanning electron microscopy) characterization of the biocomposites.^181^ The poor interfacial adhesion between the coir fiber and PBS matrix could create a gap and agglomeration during tensile strength testing for pulling out of the fibers from the matrix.^91^ However, the pretreatment of coir fiber could overcome such problems and provide better compatibility between the fiber and the matrix, thus providing better mechanical performance. If the fibers are not treated, the interfacial region of the coir fiber-based composites exhibits less compatibility, hence the composite can easily collapse.^91^ Yan *et al.*^182^ claimed that 5% alkaline treatment with NaOH for 30 min at 20 °C provided a rough but cleaner surface as displayed through SEM analysis on coir fiber-reinforced polymeric or cementitious composite panels. The failure surface of the coir fiber/epoxy composite is shown in Fig. 7(a–d) before and after the treatment across the direction of the applied load. However, treated fractured surfaces exhibited more pull-out of failed fibers than the untreated fiber composites Fig. 7(c and d). The alkali treatment of coir fiber enhances the fiber to matrix interfacial bonding, which leads to better tensile performances of biocomposites. The incorporation of more fiber volume in biocomposites could minimize the strain fracture, as the increased fillers lead to a decreased matrix quantity needed for elongation.^183^

### 5.6 Physical properties

Water absorption and thickness swelling are two very important tests for assessing the dimensional stability of biocomposites. Natural fibers absorb water from the surrounding environment or even in direct contact with the water and consequently, swelling occurs.^185^ In this regard, it is important to investigate the water absorption properties of coir fiber composites to ensure better serviceability during their usage. Water absorption has a positive relationship with the fiber length; if the length is longer, then the water absorption is higher.^186^ In general, the void content and composite density significantly affect water absorption. The greater fiber volume in the biocomposite is also responsible for greater water absorption. Biocomposites made with 20 wt% coir provided greater water absorption than 5 wt% coir fiber.^186^ The reason behind this may be that coir fiber contains hydrophilic –OH groups, as seen in the FTIR study, hence the level of moisture absorption is also high. It could therefore be concluded that increased fiber loading also increases the number of –OH groups in the composites, thus the water absorption is also increased. However, the pretreatment of coir fiber could minimize the water absorption from associated composites as the treatment reduces the –OH groups from the fibers as compared to the control.^133^

### 5.7 Thermal properties

**Fig. 8:**
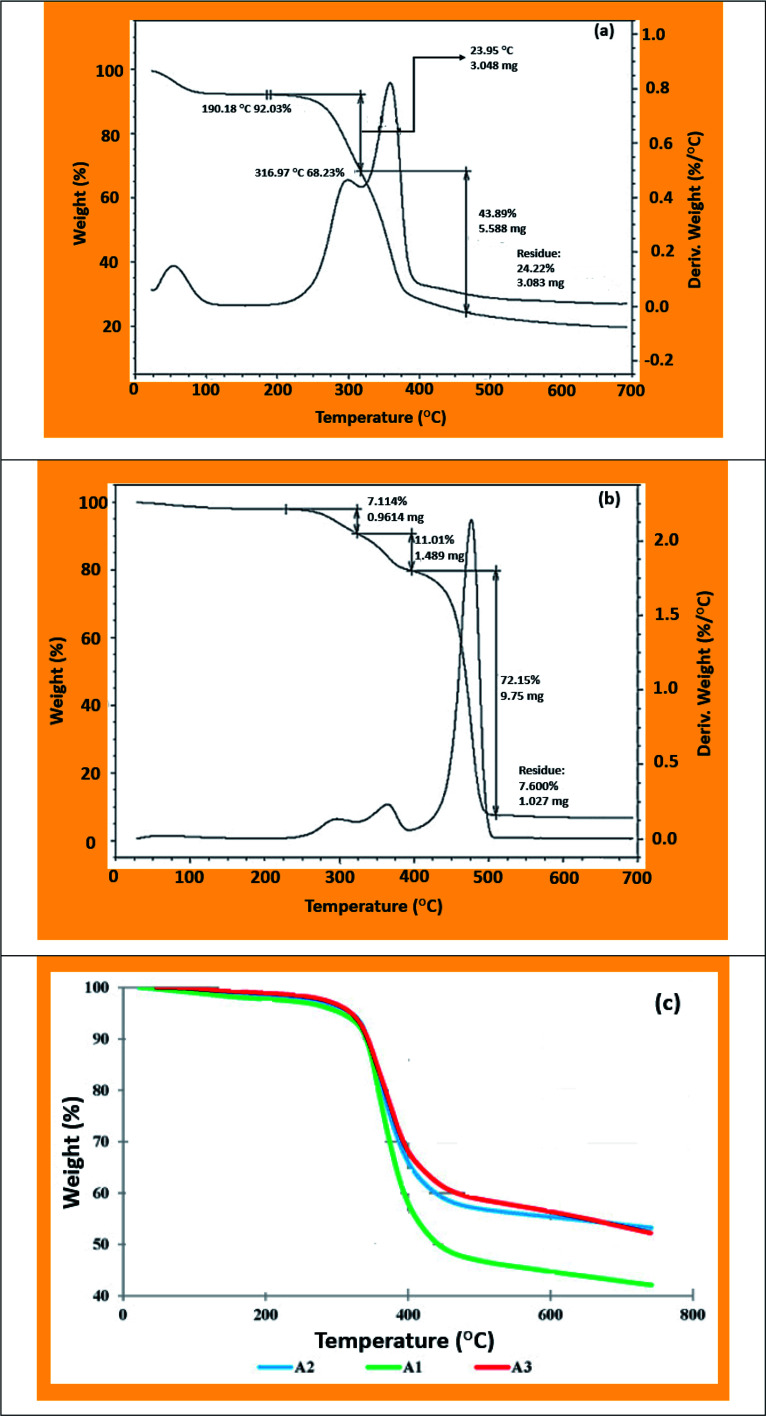
(a) TGA analysis of coir, (b) TGA analysis of coir/PP composites, and (c) TGA curve for different loadings of coir (10, 20, and 30%) with constant carbon fiber, hardener, and epoxy resin. Adapted with permission from Elsevier. Copyright, Elsevier, 2012 and 2020.^179,190^

Thermogravimetric analysis (TGA) is a useful method for investigating the weight loss of biocomposite materials corresponding to different temperatures. The structural compositions of coir fibers (lignin, cellulose, and hemicellulose) are responsible for thermal degradation due to the sensitivity to temperature.^105^ The composition of biocomposites in terms of coir and matrix along with degradation behavior could be investigated by TGA analysis. Besides, the magnitude of peaks through derivative thermogravimetric (DTG) analysis could further provide the mutual effects of components in composite systems with respect to temperature. A typical mass loss curve for a coir fiber-reinforced PP composite is illustrated in Fig. 8. The initial mass loss from room temperature (25 °C) to 150 °C is associated with water or moisture evaporations from the biocomposite panels.^187^ The initial decomposition temperature for coir fiber was observed at 190.18 °C, whereas the coir fiber/PP biocomposite exhibited decomposition at 211.2 °C, which indicates that the incorporation of PP increased the thermal stability of the composite panels. The degradation of different polymers is indicated by the mass loss at certain temperatures: the degradation of hemicellulose occurred at 200–260 °C, cellulose at 240–350 °C, and lignin at 280–500 °C.^187–189^ However, the decomposition mass loss was 23.95 and 43.89% (Fig. 8) at 190.2–316.9 °C and 316.9–475 °C, exhibiting nearly the same behaviour. Some researchers also mentioned that the pretreatment of coir fibers could also enhance the thermal stability of the biocomposites.^187^ Singh *et al.* developed coir/carbon fiber/epoxy composites; the coir fibers were 10, 20, and 30%, and the epoxy, hardener, and carbon materials were kept constant.^179^ This study claimed that the incorporation of carbon fiber and the treatment of coir fibers increased the thermal stability in composite systems and weight loss became greater with the increased coir fiber content Fig. 8(c).

### 5.8 Flame retardancy

Flammability characteristics are very important parameters for coir fiber-reinforced biocomposites, and the manufacturing of panels with improved resistance/inhibition against fire could enhance the market potential. The use of commercial fire retardants could also enhance the flame retardancy of biocomposite materials. The commercially available fire retardants are based on phosphate, nitrogen, halogen, and inorganic substances.^40^ The main purpose of a fire retardant is to inhibit the fire from reinforced composites, and they function differently depending on the physical or chemical nature of the products in the solid, gas or liquid states.^191^ It is reported that nitrogen and phosphorus-based fire retardants generate very strong effects on lignocellulosic materials. The fire retardants from boron-based compounds do not influence the mechanical properties of biocomposite materials but resist decay.^40^ Shukor *et al.*^192^ conducted a study on flame retardancy in terms of measuring limiting oxygen index (LOI) as per the ASTM D 2863 standard and found satisfactory results ranging from 28.0 to 29.4. In another report, Jang *et al.*^193^ assessed the flammability characteristics of coir fiber-reinforced PLA composites and found that all the developed composite provided LOI values higher than 20. It was mentioned by previous researchers that LOI values higher than 20 are considered non-flammable materials.^194,195^ However, Jang *et al.*^193^ has further claimed that treating the coir fibers could slightly enhance the LOI values of the composites.

## 6. Potential applications

**Fig. 9:**
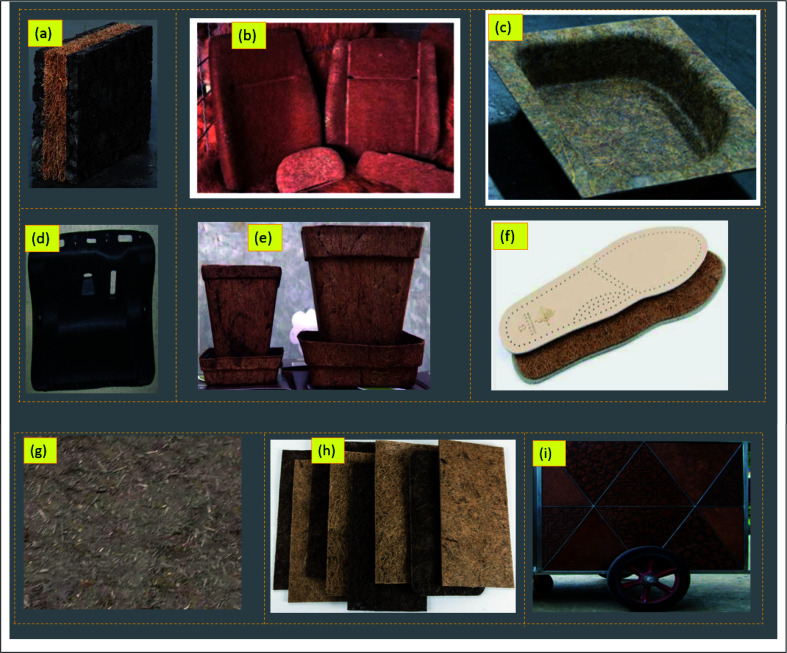
Photographs of different coconut materials-derived products: (a) thermal acoustic insulation board,^203^ (b) coir fiber seat cushion used by Mercedes Benz,^204^ (c) thermoplastic polymer-reinforced coir fiber composites,^205^ (d) truck cabin part made from coir fiber-reinforced PP,^206^ (e) pots made of coir,^207^ (f) coir-based insole,^208^ (g) coir-cement fiberboard, (h)^209^ various coconut composite panels, (i) movable coconut composite boards.^210^

Coir fibers have a long-term tradition of usage in different application areas. For a long time, coir fibers have been used as ropes, yarns, mats, floor furnishings, sackings, insulation panels, and geotextiles.^101^ However, coir fiber is showing new potential in terms of commercial prospects for manufacturing sustainable and green composite products. The light-weight, low-cost, and thermally conductive biocomposite panels are new and innovative additions of coir fiber-reinforced composites. The coir-reinforced fibers are widely used for composite panels, beams, and slabs.^196^ Besides, coir-fiber-based composites also show tremendous potential for seat cushioning in the automotive and construction sectors.^187,197^ A funnel developed from coir-based materials provides good dimensional stability and mechanical strength.^198^ The same study also reported flower pots having high water retention properties made of coir reinforced PP biocomposites.^198^ Coir pith could also be used as lightweight and non-structural building materials through reinforcement with a cementitious matrix, providing thermal and acoustic performances with 3.97–4.35 MPa compressive strength and 0.99–1.26 g cm^−3^ bulk density.^199^ Luz *et al.* developed a multilayered armor system by using 30% coir fiber reinforced with epoxy resin to produce composite materials for ballistic performances.^200^ They further claimed that the reported composite displayed similar performances to Kevlar-based materials.^200^ The biocomposite materials made from coir/PP could be used for automotive parts.^126^ Nadir *et al.* developed composite panels by reinforcing coir with PP for automotive interiors.^201^ Coir-based materials could further be used as helmets, post-office boxes, and roofing materials.^26^ Different commercially available and ongoing research-based biocomposite products from coir fibers are shown in Fig. 9. Coir fiber also shows tremendous application potential in the fields of furniture, aerospace (propellers, wings, and tails), boat hulls, sporting goods, cementitious particle boards, and packaging.^31,202^

## 7. Economical aspects and environmental sustainability of coir fiber-reinforced composites

The global composite market is booming with the continuously increasing demands of consumers. The world composite market was projected to be USD 74 billion by 2020, whereas this figure could be enhanced up to USD 112.8 billion by 2025 with an 8.8% compound annual growth rate (CAGR).^211^ However, with the constantly increasing environmental awareness of the people, synthetic material-based composites are being replaced by biocomposite materials. The market volume of biocomposite in 2016 was USD 16.46 billion, whereas it was projected to be 36.76 billion by 2022 with a 14.44% CAGR from 2017.^212^ The biocomposite market is still an untapped sector where there is the potential for a huge market with prominent demands. The biocomposite products are gaining tremendous attention from the aerospace, automotive, consumer and sporting goods, packaging, biomedical, and construction sectors. There are lots of efforts being made to explore more export-oriented coir fiber and its associated markets, as most coir fibers remain underutilized. In contrast to the potential competitiveness, the progress in the production of coir fiber-reinforced biocomposites and associated employment generations is still low or constant. Coir-based industries are also facilitating huge employments from coconut cultivations to fiber extractions, and associated biocomposite production. The total production of coir fibers is 350 000 metric tonnes annually throughout the world.^213^ Compared to other natural fibers, coir fiber and associated materials also contribute significantly to biocomposite markets. Coir fibers occupied around $369.7 million by 2019 which is expected to reach $525.7 million by 2027 with an 8.2% CAGR rate within this period.^214^ However, due to constant demands for coir-based materials, there is an expansion in USA and Europe.

The processing associated with coir fiber, like retting, is a major issue for generating pollutants.^215^ Coconut husk retting in India is traditionally performed in water systems for 6 to 12 months long durations, which is an age-old process to extract coir fibers. A large number of organic chemical substances like tannin, pectin, fat, phenolic compounds (toxic), and pentosans from coconut husks are liberated in the water systems.^215^ Such retting processes of coconut husks also affect the living space of aquatic living agents like fish and also impact the tidal force of water sources. Besides, the air of the surrounding area of retting is affected by a bad smell that pollutes the surrounding atmosphere. The biological retting process for coconut husks is little bit different as compared to other natural fibers like jute, as only pectin is decomposed from jute but beside the pectin, phenolic compounds are also decomposed and disintegrated from coir. The pectinolytic action of microorganisms like bacteria, yeast, and fungi degrades the fiber-binding elements from husks and liberates them into the environment in large amounts, in terms of organic chemicals and materials. As the DO (dissolved oxygen) is decreased, the hydrogen sulphide, nitrate, and phosphate contents are increased as a consequence of retting-related waste generations in water systems. However, different studies are also trying to find alternative routes for retting processes to eliminate such environmental challenges.^53^ Recently, some of the manufacturers were also trying to treat coir fibers with bleaching and scouring chemicals for fiber-to-matrix adhesion improvements or coloration purposes to meet the demands of consumers; hence chemical-based waste is also polluting the water sources.^59^

## 8. SWOT (strengths, weaknesses, opportunities, and threats) analysis of coir fibers and associated biocomposites

### 8.1 Strengths

- Potential biodegradability feature

- Awareness of sustainability throughout the world

- Constantly increasing demands toward natural fiber and associated byproduct-reinforced biocomposites

- Lower density, higher stiffness, and higher strengths

- Economical when produced on the industrial scale

- Minimizes/eliminates hazardous effects from manufacturing operations

- Renewability and recyclability

- Requires less energy for processing

### 8.2 Weakness

- Differences in inherent characteristics

- Weaker interfacial bonding

- More feasible production technology is not yet invented

### 8.3 Opportunity

- Demands on eco-friendly sustainable products are increasing

- Demands for a lightweight biocomposite material is high

- Researchers and manufacturers are paying more attention to natural fiber-reinforced biocomposites

- Biocomposite manufacturing is also implementing state-of-the-art technology with improved scientific inventions and knowledge

- Manufacturers are trying to be more sustainable to cope with more customer demands

### 8.4 Threats

- Climate change is having a critical impact, affecting the availability of raw materials (plant-based) all over the world

- Each specialized application needs specific high-performance fibers

- The cheaper price of synthetic materials

- Non-homogeneous quality of the natural fibers

## 9. Conclusion

This study has provided an overall discussion on coir fiber as a potential filler material for producing biocomposite panels. The physical, chemical, morphological, thermal, and mechanical properties of coir fiber materials, which affect the ultimate biocomposite features, have also been discussed in this review. The surface modifications of natural fibers like coir could also play a significant role in the mechanical properties of biocomposite materials through improving the interfacial adhesion between the coir and matrix, which has been addressed. It was found that the mechanical properties of coir fiber-based composites are dependent on the matrix used. Besides, the –OH groups of the treated coir materials decrease during the pretreatment processes; hence the untreated fibers absorb more moisture in contrast to the treated fibers. Another promising finding reported that the pretreatment could facilitate the reduction of the void content, which is a challenging problem in manufacturing biocomposites. However, the coir fibers are suitable for particle boards, structural beams, thermal insulation, sound absorption panels, and so on. The coir fiber-reinforced biocomposites also provide excellent mechanical performances and thermal stability. The hybrid composite materials developed from coir fiber and other natural or synthetic fibers could tune the improved thermo-mechanical performances of the composites. Furthermore, with the expansion of scientific innovations and technology, there are more areas of coir fiber-reinforced composite applications, which also influences the constantly increasing market for this emerging material. Further investigation is necessary to develop the coir fiber-based composites from all the possible polymeric matrixes and dynamic characteristics like damping ratio and natural frequency.

## Abbreviations

CO_2_: Carbon dioxide
PP: Polypropylene
H_2_SO_4_: Sulphuric acid
NaHCO_3_: Sodium bicarbonate
CrSO_4_: Chromium sulphate
Na_2_CO_3_: Sodium carbonate
NaOH: Sodium hydroxide
MAPP: Maleic anhydride grafted polypropylene
CH_3_CO: Acetic anhydride
3D: Three-dimensional
PLA: Polylactic acid
PE: Polyethylene
HDPE: High density polyethylene
PES: Polyester
MUF: Melamine urea formaldehyde
RTM: Resin transfer molding
ASTM: American society for testing and materials
MOE: Modulus of elasticity
TS: Tensile strength
MOR: Modulus of rupture
*ρ*
: Density
TM: Tensile modulus
IBS: Internal bonding strength
IS: Impact strength
ThS: Thickness swelling
WA: Water absorbency
FM: Flexural modulus
EB: Elongation at break
PBS: Polybutylene succinate
SEM: Scanning electron microscope
TGA: Thermogravimetric analysis
DTG: Derivative thermogravimetric
LOI: Limiting oxygen index
CAGR: Compound annual growth rate
DO: Dissolved oxygen
SWOT: Strengths, weaknesses, opportunities, and threats

## Author contributions

The manuscript has been written with the contribution of all the authors. All the authors are agreed on the submitted final version of this manuscript.

## Conflicts of interest

The authors have no conflict of interest.

## Supplementary Material
